# The Thermal Stability of Influenza Viruses in Milk

**DOI:** 10.3390/v16111766

**Published:** 2024-11-13

**Authors:** Wanke Hu, Zhao Wang, Yunxia Chen, Siyu Wu, Tianyu Li, Shao-Lun Zhai, Xianghong Ju, Yipeng Sun, Wen-Kang Wei, Jieshi Yu

**Affiliations:** 1 College of Coastal Agricultural Sciences, Guangdong Ocean University, Zhanjiang 524088, China; 2112204099@stu.gdou.edu.cn (W.H.); juxh77@163.com (X.J.); 2 State Key Laboratory of Swine and Poultry Breeding Industry, Agro-Biological Gene Research Center, Guangdong Academy of Agricultural Sciences, Guangzhou 510640, China; yunxiachen22@163.com (Y.C.); wusiyu@gdaas.cn (S.W.); 3 School of Laboratory Animal, Shandong First Medical University, Jinan 250117, China; zhaowang2011@foxmail.com; 4 College of Veterinary Medicine, South China Agricultural University, Guangzhou 510642, China; 5 Zhongshan Animal Disease Control Center, Zhongshan 528400, China; lipai513@163.com; 6 Key Laboratory of Livestock Disease Prevention of Guangdong Province, Institute of Animal Health, Guangdong Academy of Agricultural Sciences, Guangzhou 510640, China; zhaishaolun@163.com; 7 National Key Laboratory of Veterinary Public Health and Safety, Key Laboratory for Prevention and Control of Avian Influenza and Other Major Poultry Diseases of Ministry of Agriculture and Rural Affairs, College of Veterinary Medicine, China Agricultural University, Beijing 100193, China; sypcau@163.com

**Keywords:** influenza virus, milk, pasteurization, inactivation

## Abstract

Highly pathogenic avian influenza viruses (HPAIVs) of the H5N1 subtype (clade 2.3.4.4b) have been detected in raw milk from infected cows. Several studies have examined the time and temperature parameters to ascertain whether influenza viruses in milk can be inactivated completely under commercial pasteurization conditions, yielding conflicting results. This study aimed to investigate whether milk could help protect influenza viruses from heat treatment. After heat treatment at 49 °C for one hour, the titer reduction of the influenza A/WSN/1933 (A/H1) virus in milk was approximately 1.6 log_10_TCID_50_/mL, which was significantly lower than that (3 log_10_TCID_50_/mL) observed in the Dulbecco’s Modified Eagle Medium (DMEM) control media. The influenza D/bovine/CHN/JY3002/2022 (D/Yama2019) virus in milk retained a high residual infectivity (4.68 × 10^3^ log10TCID_50_/mL) after treatment at 53 °C; however, the virus in DMEM completely lost its infectivity under the same conditions. Moreover, the influenza A/chicken/CHN/Cangzhou03/2023 (A/H5) virus in DMEM could be inactivated completely using any of the three heat treatment methods: 63 °C for 30 min, 72 °C for 15 s, or 80 °C for 15 s. For the virus present in milk, only heat treatment at 80 °C for 15 s completely inactivated it. These results suggest that milk prevents influenza viruses from pasteurization inactivation.

## 1. Introduction

Highly pathogenic avian influenza viruses (HPAIVs) of the H5N1 subtype (clade 2.3.4.4b) have spread widely among dairy cattle in the United States [1,2]. Unexpectedly, high levels of viable H5N1 HPAIVs have been detected in raw milk from those infected cows [3,4]. It has raised serious concerns regarding the safety of the cow’s milk supply [5,6]. Pasteurization, a heat-treatment process to eliminate pathogens, ensures the safety of commercial milk. However, recent studies have shown inconsistent results when simulating pasteurization conditions to inactivate the virus in contaminated milk.

One study found that heat treatment of H5N1 HPAIV-positive milk samples at 63 °C for 5 to 30 min or at 72 °C for 15 or 20 s reduced the virus titers below the detection limit that was conducted in Madin–Darby canine kidney (MDCK) cells with the traditional 50% tissue-culture infectious dose (TCID_50_) as the readout [3]. Nevertheless, this study also reported that heat treatment of H5N1 HPAIV-positive milk samples at 72 °C for 15 or 20 s did not completely disarm the virus, as the inoculation of the treated samples into embryonated chicken eggs still resulted in detectable infectious virus particles [3]. Despite a similar conclusion, another independent study noticed that if initial titers for H5N1 HPAIVs were substantially higher, relatively small amounts of the virus remained infectious in milk even following 15 s of treatment at 72 °C, as determined by the TCID_50_ assay in MDCK cells [7]. The latest study examining the effects of pasteurization-like temperatures on influenza viruses in retail and unpasteurized milk showed that heat treatment at 72 °C for 20 s in a 20 µL sample volume significantly reduced influenza virus titer from ~10^8^ TCID_50_/mL to ~10^4^ TCID_50_/mL [8], which implied incomplete killing of influenza viruses under such conditions. However, this study revealed that heat treatment at 63 °C for 30 min could effectively reduce influenza virus viability below the detection limit in MDCK cells [8]. Similarly, another recent study demonstrated that H5N1 HPAIVs could be completely inactivated by incubation of the virus-spiked raw milk at 63 °C for 30 min, while virus inactivation was achieved in seven out of eight experimental replicates when the virus-spiked raw milk was subjected to treatment at 72 °C for 15 s [9]. In addition, a separate study showed the complete inactivation of an H5 virus in raw milk after treatment at 72 °C for 15 s in a polymerase chain reaction (PCR) thermocycler [10]. Moreover, another recent study reported that no viable virus could be detected when HPAIV-artificially-contaminated raw milk was treated under closely approximating commercial milk-pasteurization conditions [11].

These divergent results highlight the need for further investigation to elucidate the impact of heat treatment on the inactivation of influenza viruses. In the present study, we investigated the thermal stability of influenza viruses in artificially contaminated milk and Dulbecco’s Modified Eagle Medium (DMEM) control media. In addition to HPAIV H5N1 and influenza A/WSN/1933 viruses (Genus: *Alphainfluenzavirus*, Species: *Alphainfluenzavirus influenzae*, influenza A virus), we also include an influenza D virus (Genus: *Deltainfluenzavirus*, Species: *Deltainfluenzavirus influenzae*) strain with bovine as a primary reservoir.

## 2. Materials and Methods

Cells and viruses. MDCK cells were maintained at 37 °C with 5% CO_2_. High-glucose DMEM (Cytiva HyClone, Logan, UT, USA) supplemented with 10% (*v*/*v*) fetal bovine serum (FBS, Procell) and 100 U/mL penicillin-streptomycin (Life Technologies, Carlsbad, CA, USA) was used to culture MDCK cells. The human influenza A/WSN/1933 (A/H1) virus was rescued by the reverse genetics system [12], influenza A/chicken/CHN/Cangzhou/2023 (A/H5) virus (GenBank: PQ278123-PQ278130) was isolated from chicken by inoculation of a clinical sample into embryonated chicken eggs, and influenza D/bovine/CHN/JY3002/2022 virus was isolated from cattle and stored in the laboratory [13]. These viruses were propagated in MDCK cells or embryonated chicken eggs. The experiments involving the influenza A/H5 virus were carried out in the Biosafety Level 3 laboratory.

Heat treatment. The commercial pasteurized whole-fat milk (Member’s Mark, Sam’s Club, Guangzhou, China) used in this study contains 3.8% fat, 3.2% protein, and 5% carbohydrates. Before heat treatment, a volume of influenza A/H1, A/H5, or D/Yama2019 virus was mixed with three volumes of milk to make the artificially contaminated milk or three volumes of DMEM to serve as a control. The mixture was aliquoted into 12.5 µL or 50 µL per tube and then incubated under different temperatures (4 °C, 37 °C, 49 °C, 53 °C, and 57 °C) or pasteurized with different procedures (63 °C for 30 min, 72 °C for 15 s, or 80 °C for 15 s) in a PCR thermocycler (Bio-Rad, T100 Thermal Cycler, Foster City, CA, USA). After incubation for indicated times at 4 °C, each virus solution in milk or DMEM was titered by TCID_50_ assays. The influenza A/H1 or D/Yama2019 virus in milk or DMEM was treated under 37 °C, 49 °C, 53 °C, and 57 °C for one hour and incubated for another half hour in 4 °C prior to the TCID_50_ titration. The influenza A/H1, A/H5, or D/Yama2019 virus in milk or DMEM was heat treated at 63 °C for 30 min, 72 °C for 15 s, or 80 °C for 15 s in the PCR thermocycler. Briefly, the aliquoted sample (12.5 µL or 50 µL per PCR tube) was incubated at room temperature for 10 min. At the same time, the thermocycler lid was preheated to the temperature for treatment and then set procedures as follows: (1) 25 °C for 45 s, (2) 63 °C for 30 min, 72 °C for 15 s, or 80 °C for 15 s, and (3) 4 °C for 5 min. Samples in PCR vials were placed into the thermocycler and treated by running the indicated procedures. The treated samples were removed from the machine and immediately placed on ice. Infectious viruses in heat-treated and untreated samples were measured by the TCID_50_ assays or tested by inoculation into embryonated chicken eggs. For testing in embryonated chicken eggs, a mixture of two tubes of pasteurized samples with a reaction volume of 50 µL or a mixture of eight tubes of pasteurized samples containing a reaction volume of 12.5 µL was prepared prior to inoculation. The prepared mixtures were then inoculated into embryonated chicken eggs to detect the survival virus. Upon death, eggs were tested for hemagglutination titers (HA). The remaining live eggs were also tested four days after inoculation.

Statistical analysis. The significant differences between different groups were determined by the Student’s t-test or the one-way ANOVA followed by Tukey’s multiple comparison test in GraphPad Prism 8.0. Values of *p* < 0.05 were considered significant (* *p* < 0.05; ** *p* < 0.01; *** *p* < 0.001; **** *p* < 0.0001).

## 3. Results

**Figure 1:**
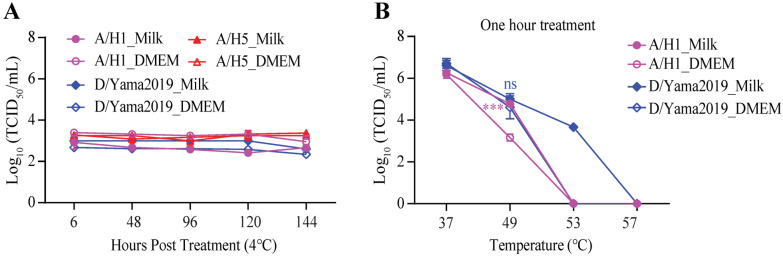
The thermal stability of influenza viruses in milk. A volume of influenza A/WSN/1933 (A/H1), A/chicken/CHN/Cangzhou/2023 (A/H5), or D/bovine/CHN/JY3002/2022 (D/Yama2019) virus was added to three volumes of milk to make the artificially contaminated milk or to three volumes of DMEM to serve as a control. (**A**) The influenza A/H1, A/H5, or D/Yama2019 virus in milk or DMEM was diluted to a low titer (~4 × 10^3^ TCID_50_/mL). After incubation for the indicated time at 4 °C, the virus solution was titered by the TCID_50_ assays. (**B**) The influenza A/H1 or D/Yama2019 virus in milk or DMEM was treated under different temperatures (37 °C, 49 °C, 53 °C, and 57 °C) for one hour and incubated for another half hour at 4 °C prior to the TCID_50_ titration. Error bars represent the standard error of the mean (SEM) for at least three independent experiments. ns: not significant; *** *p* < 0.001.

One volume of a low titer (~4 × 10^3^ TCID_50_/mL) of influenza A/WSN/1933 (A/H1), A/chicken/CHN/Cangzhou03/2023 (A/H5), or D/bovine/CHN/JY3002/2022 (D/Yama2019) was mixed with three volumes of commercial whole-fat milk or DMEM, respectively. There was no significant decline in virus titers over one week of storage at 4 °C (Figure 1A). These results demonstrated the stability of influenza viruses in milk or in DMEM held at refrigerated temperatures. To determine if milk could protect the virus against the heat treatment, the A/H1 (1.66 × 10^6^ TCID_50_/mL) or D/Yama2019 (4.57 × 10^6^ TCID_50_/mL) in milk or DMEM were treated at 37 °C, 49 °C, 53 °C, and 57 °C for one hour, respectively, followed by another half hour incubation on ice. The residual titer of A/H1 in milk was ~1.6 log_10_TCID_50_/mL higher than that in DMEM when treated at 49 °C, while the A/H1 in milk or DMEM was completely inactivated when treated at 53 °C (Figure 1B). The D/Yama2019 in milk retained a high residual infectivity (4.68 × 10^3^ log_10_TCID_50_/mL) after treatment at 53 °C, but the D/Yama2019 in DMEM completely lost its infectivity after treatment under the same condition (Figure 1B). These results indicated that milk provided protection against influenza viruses during heat treatment.

**Figure 2:**
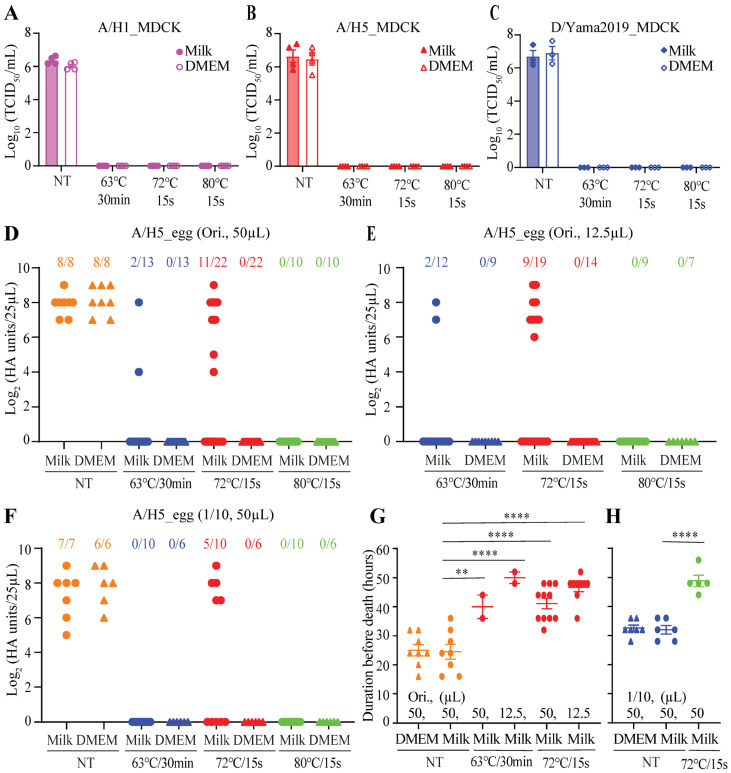
Heat treatment of influenza viruses in milk. (**A**–**F**) The influenza A/H1, A/H5, or D/Yama2019 virus in milk or DMEM was heat treated with three different procedures (63 °C for 30 min, 72 °C for 15 s, or 80 °C for 15 s) in a PCR thermocycler. Specifically, the sample was aliquoted into 12.5 µL (**E**) or 50 µL (**A**–**D**,**F**) per PCR tube and incubated at room temperature for 10 min. Meanwhile, the thermocycler lid was preheated to the temperature for treatment and then set procedures as follows: (1) 25 °C for 45 s, (2) 63 °C for 30 min, 72 °C for 15 s, or 80 °C for 15 s, and (3) 4 °C for 5 min. Samples in PCR vials were placed into the thermocycler and treated by running the indicated procedures. The treated samples were removed from the machine and immediately placed on ice. Infectious viruses in heat-treated and untreated samples were measured by TCID_50_ assays (**A**–**C**) or tested by inoculation into embryonated chicken eggs (**D**–**F**). “Ori., 50 µL” and “Ori., 12.5 µL” represented that original virus solution was used and pasteurized with a reaction volume of 50 µL and 12.5 µL, respectively. Similarly, “1/10, 50 µL” represented that 1/10 diluted virus solution was used and pasteurized with a reaction volume of 50 µL. Numbers with different colors at the top of panels D-F, such as “11/22”, represented that 11 out of 22 inoculated eggs were dead due to A/H5 infection. (**G**,**H**) For eggs infected with viable A/H5, the duration of each egg before death was recorded. Error bars represent the standard error of the mean (SEM) for at least three independent experiments. ** *p* < 0.01; **** *p* < 0.0001.

One volume of the virus was added to three volumes of milk or DMEM to evaluate the effect of thermal pasteurization on the inactivation of influenza viruses in milk, and the mixture was aliquoted into 12.5 µL or 50 µL per tube, which was then pasteurized with three different procedures in a PCR thermocycler. Regardless of the pasteurization procedures, virus strains, and mixing with milk or DMEM, all treatments resulted in no detectable viruses when assayed using the MDCK-based TCID_50_ experiments (Figure 2A–C). Since the A/H5 virus was isolated from chicken and its titer in embryonated chicken eggs (1.91 × 10^8^ EID_50_/mL) (50% egg infectious dose per milliliter) was higher than that measured in MDCK cells (3.55 × 10^6^ TCID_50_/mL), pasteurized samples were also inoculated into embryonated chicken eggs for detecting survival of viruses.

After heating, each virus-contaminated milk sample at 63 °C for 30 min, 2 out of 13 samples with a reaction volume of 50 µL (Figure 2D) and 2 out of 12 samples with a reaction volume of 12.5 µL (Figure 2E) retained viable viruses. Notably, 11 out of 22 samples with a reaction volume of 50 µL (Figure 2D) and 9 out of 19 samples with a reaction volume of 12.5 µL (Figure 2E) retained infectious virus particles when virus-contaminated milk samples were treated at 72 °C for 15 s. Under treatment at 72 °C for 15 s (reaction volume: 50 µL), virus survival was also found in 5 out of 10 milk samples containing 1/10 diluted A/H5 (Figure 2F). In milk, only 80 °C for 15 s destroyed the A/H5 virus completely, while in DMEM, all three heat treatment procedures inactivated the virus completely (Figure 2D–F). In dead eggs, recovered HA titers of heat-treated virus samples were relatively lower or comparable to HA titers of non-treated virus samples (Figure 2D–F), while it took treated virus samples significantly longer to cause egg death (Figure 2G,H). These results further suggested that milk could protect influenza viruses against heat inactivation, and heat treatment at 80 °C for 15 s was superior to simulated pasteurization at 72 °C for 15 s or at 63 °C for 30 min in fully killing the virus in milk.

## 4. Discussion

There is widespread recognition that pasteurization of raw milk inactivates potential pathogens. Due to the recent discovery of HPAIV H5N1 in cattle and the observation of virus tropism in the mammary gland [1,2,14], a greater focus is being given to determining which parameters are necessary to specifically inactivate HPAIV H5N1 in milk. Published studies displayed variable results of using pasteurization-like conditions to inactivate influenza viruses present in contaminated milk. Several factors may contribute to the varying results. Two studies [3,10], plus this study, were conducted utilizing PCR thermocyclers with similar reaction volumes (10 to 12.5 µL). Our results (Figure 2E) and the results [3] from one of the two studies suggested that pasteurization at 72 °C for 15 s could not fully kill HPAIV H5N1 in milk. However, another of the two studies found complete inactivation of HPAIV H5N1 in milk at 72 °C for 15 s [10]. Differences in the pre-incubation of the sample, the pre-heating of the thermocycler block, the post-treatment incubation of the sample, and the volume of the sample inoculated into the embryonated chicken egg might lead to different outcomes. Three studies [7,8,9] were performed using a dry bath with different reaction volumes (e.g., 200 µL, 750 µL, 1mL). These studies indicated that HPAI H5N1 remained infectious in milk after 15 or 20 s treatment at 72 °C. [7,8,9]. One study [11] used a modified pilot-scale continuous-flow pasteurizer to evaluate HPAIV inactivation in artificially contaminated raw milk. It showed that the entire inactivation of ~1 × 10^6^ EID_50_/mL HPAI H5N1 could be achieved during the 7.2 s of ramped heating from 63 °C to 72.5 °C [11]. The heating efficiency of flowing milk in a coiled tube [11] may be higher than that of milk in a standing tube [3,7,8,9,10], which warrants further investigation. In addition, the virus concentration employed for heat treatment in our study, as well as in one other study [8] (>1 × 10^8^ EID_50_/mL) that showed a greater quantity of residual viable virus in the heat-treated milk, was higher than that used in the other studies (<1 × 10^8^ EID_50_/mL) [3,7,9,10,11].

None of the currently published studies demonstrated that milk could protect influenza viruses against heat inactivation. The data from this study provide convincing evidence that milk increases influenza viruses’ thermal resistance. Influenza A/H1, A/H5, and D/Yama2019 viruses used in the study displayed greater survivability against heat treatment in milk than in DMEM (Figure 1B and Figure 2D–F). Milk compositions probably protect viruses from inactivation by pasteurization [15]. This might make milk products with a high level of fat, protein, and/or sugar content particularly resistant to virus inactivation. A recent study found that the bovine influenza H5N1 virus stayed active in a concentrated lactose solution for up to 14 days under refrigerated conditions [16]. Fortunately, heat treatment at 66 °C for a minimum of five minutes could efficiently inactivate the virus in lactose [16].

This work also emphasizes that various variables, including virus strains, cell-free or cell-associated status of the virus, and survival virus detection methods, should be considered when using a heat inactivation experiment to evaluate the impact of temperature on the stability of influenza viruses, including HPAIV H5N1 circulated in dairy cows. Both this (Figure 2B,D–F) and a recent publication [3] showed that residual viruses post-pasteurization could not be detected using MDCK cells-based TCID_50_ tests but can be recovered by inoculating embryonated chicken eggs. A sensitive and efficient measuring method might be necessary to determine whether the remaining viable virus was present.

This study observed that heat treatment at 80 °C for 15 s could completely inactivate HPAIV H5N1 in milk (Figure 2D–F). Neither pasteurization at 72 °C for 15 s nor at 63 °C for 30 min could fully kill the virus in milk (Figure 2D–F). The published studies showed that pasteurization at 63 °C for 30 min could effectively reduce viral viability below the limit of detection, but viable viruses could still be detected after pasteurization at 72 °C for 15 or 20 s [3,7,8,9]. Typically, milk safety is enhanced by higher heat treatment temperatures and/or longer treatment times. However, milk quality and taste are also crucial in the dairy industry and may be compromised by excessive treatment temperatures and times.

This study has several limitations. First, although we have demonstrated that milk can protect influenza viruses from heat inactivation, the potential mechanisms underlying this protection have not been elucidated. Components of milk, such as fat, protein, and sugar, likely contribute to this protective effect, which warrants further studies. Second, although we have proven that simulated pasteurization at 72 °C for 15 s or at 63 °C for 30 min cannot fully kill influenza viruses in milk, these findings do not necessarily indicate that the same results occur in commercial pasteurization. Actually, commercial milk pasteurization processing at high temperatures for a short time has a pre-heating step to ensure milk enters the final heater at a consistent temperature of 37.8 °C, a 7.2-s ramped heating from 63 °C to 72.5 °C, and a 15-s holding time at a temperature of 72 °C [11]. However, in addition to our study, at least three other studies [3,7,8] clearly indicate that simulative pasteurization at 72 °C for 15 s does not completely inactivate the influenza virus in milk. Only one study, which closely approximates commercial milk pasteurization, indicates that high temperature for a short time pasteurization is 100% effective in inactivating the influenza virus in milk [11]. Moreover, our study confirmed that milk could protect influenza viruses from heat inactivation. Therefore, future studies using closely approximating commercial milk pasteurization with raw milk from infected cows are needed to demonstrate the safety of commercially pasteurized milk.

## Data Availability

Data will be made available on request.
